# The importance of delineating networks by activity type in bottlenose dolphins (*Tursiops truncatus*) in Cedar Key, Florida

**DOI:** 10.1098/rsos.140263

**Published:** 2015-03-11

**Authors:** Stefanie Gazda, Swami Iyer, Timothy Killingback, Richard Connor, Solange Brault

**Affiliations:** 1 Department of Biology, University of Massachusetts, Boston, MA 02125, USA; 2 Department of Computer Science, University of Massachusetts, Boston, MA 02125, USA; 3 Department of Mathematics, University of Massachusetts, Boston, MA 02125, USA; 4 Department of Biology, University of Massachusetts, Dartmouth, MA 02747, USA

**Keywords:** bottlenose dolphin, behaviour, network

## Abstract

Network analysis has proved to be a valuable tool for studying the behavioural patterns of complex social animals. Often such studies either do not distinguish between different behavioural states of the organisms or simply focus attention on a single behavioural state to the exclusion of all others. In either of these approaches it is impossible to ascertain how the behavioural patterns of individuals depend on the type of activity they are engaged in. Here we report on a network-based analysis of the behavioural associations in a population of bottlenose dolphins (*Tursiops truncatus*) in Cedar Key, Florida. We consider three distinct behavioural states—socializing, travelling and foraging—and analyse the association networks corresponding to each activity. Moreover, in constructing the different activity networks we do not simply record a spatial association between two individuals as being either present or absent, but rather quantify the degree of any association, thus allowing us to construct weighted networks describing each activity. The results of these weighted activity networks indicate that networks can reveal detailed patterns of bottlenose dolphins at the population level; dolphins socialize in large groups with preferential associations; travel in small groups with preferential associates; and spread out to forage in very small, weakly connected groups. There is some overlap in the socialize and travel networks but little overlap between the forage and other networks. This indicates that the social bonds maintained in other activities are less important as they forage on dispersed, solitary prey. The overall network, not sorted by activity, does not accurately represent any of these patterns.

## 2. Introduction

Social animals are by their very nature embedded in a network of relationships. Traditionally, in behavioural ecology these relationships are analysed at the dyadic level, i.e. at the level of pairwise relationships between individuals [1], but such an approach runs the risk of overlooking those aspects of social relationships that depend on the totality of the network of interactions in which the individuals are enmeshed [2–4]. The fluctuations in the dyadic patterns of behaviour can be well captured by network features [5]. For this reason, network analysis, which has become an important tool in understanding systems of interacting objects in many areas of biological [6,7], physical [8,9] and social sciences [10], plays a valuable role in studying the effect of complex patterns of relationships of social organisms [1–4,11–18]. Networked systems consist of a set of vertices together with a set of edges, each of which connects two vertices [19,20]. In the context of social organisms, the vertices in the network represent the individual animals, and the edges represent a connection (e.g. direct interactions, home-range overlap or as also in this study associations) between the corresponding animals [2–4].

Many network analyses of social animals [15,21,22] consider patterns of connections between individuals across all behavioural states or focus solely on associations during socializing activities. However, group members interact in different behavioural contexts, and the interactions in one state may or may not be independent of those in others [5]. Gero *et al.* [23] showed that dyadic associations between individuals can vary depending on the behavioural state considered and suggested that it may be an oversimplification to analyse complex organisms using a single network. In female northern long-eared bats, preferred associations and social network metrics vary with reproductive period [24]. Therefore, a network analysis of a population is likely to produce different results depending on the behavioural states of the animals used to construct the network.

There is an extensive literature on the fission–fusion social patterns of mammals (reviewed in [25]), where group size and composition change daily or even hourly based on the activity. Examples of such studies include allomaternal care in elephants [26], reproductive competition in male bottlenose dolphins [27–29], safety in sleeping locations in hamadryas baboons [30] and congregation in giant mouse lemurs for mating or competing for resources [31]. Most fission–fusion studies gather data on associations based on the ‘Gambit of the Group’, which assumes that everyone in a group is associating with each other [32]. Data from these observations can be combined into networks that are cumulative and then analysed for non-random features [33,34]. However, presence within a group may not always represent a real association, and observation time is often limited, so the data collected may only be a rough estimate of the entire social structure of the population. Applying a weighted association index removes some sampling bias by filtering out weak associations [32,33,35]. This is still a rough analysis of the population as it does not take the context of the associations that formed the network into account. Creating separate networks in which behaviours are the sorting factor may lead to a much more realistic portrayal of the structure and relationships within the population in question.

Group living is a trade-off between competing factors. Major reasons why individuals form groups are reduction of predation risk, increased access to resources and when the distribution of these resources promotes grouping. Groups can also reduce foraging efficiency and increase competition, among other costs (reviewed in [36]). Network analysis in species with fission–fusion grouping patterns can help develop a more complete explanation of social structure [37], where there are contrasting pressures of predator avoidance and feeding competition [25]. Social patterns across different behavioural activities of many fission–fusion populations may be optimally studied using network analysis [25]. For example, in male African elephants (*Loxodonta africana*) controlling for behavioural (sexual) state revealed more complex male-male associations than were known previously [38]. Male zebras have differing association patterns with other males depending on whether they are stallions defending a herd of females or bachelors [39]. When meerkat networks are sorted by behaviour, differences in an individual's attributes do not consistently influence association patterns across behaviour-specific networks [17].

This paper reports on a network-based study of the social patterns across three activity states (socializing, travelling and foraging) of a population of *Tursiops truncatus* located in Cedar Key, Florida. The null hypothesis is that regardless of activity state, the corresponding networks will be similar to each other and to the overall network that does not take activity into account. There are good reasons to suspect the null hypothesis might be true. For fission–fusion species that disperse to forage in bouts, costs of locomotion will greatly impact the ability to form social groups between foraging bouts [40]. Bottlenose dolphins (*Tursiops* spp.) have low locomotor costs [41] and are well known for their fission–fusion grouping patterns (reviewed in [42]). Because dolphins have such low costs of movement, grouping is much less likely to be affected by this variable. If the costs or benefits of group formation or partner preferences vary with activity, the different networks should reflect this.

Our predictions for the alternative hypotheses are as follows.

— *Socialize network.* Bottlenose dolphins are highly social animals and often have preferential associations. They express these associations with affiliative behaviours such as physical contact and synchronous movements. Socio-sexual behaviours are also common, and they do not have to be with preferred associates [42]. Thus, the socialize network should indicate some preferential associations among individuals. Moreover, dolphins that are connected to a particular dolphin are also expected to be connected to one another.
— *Travel network.* Gero *et al.* [23] showed that bottlenose dolphins in Shark Bay Australia (*T. cf. aduncus*) have weaker associations while travelling compared with socializing or foraging groups. Given that cooperation and competition are less prominent aspects of travelling compared with social and foraging interactions, this is not surprising. Accordingly, we expect Cedar Key dolphins will have less pronounced preferential travel associations, have few weak associations with others and as a result travel alone or in small, weakly connected groups. Also, dolphins that are connected to a particular dolphin are not expected to be connected to each other.
— *Forage network.* Gero *et al.* [23] demonstrated that preferred associations in the Shark Bay *T. cf. aduncus* are strongest when socializing or foraging. If this is the case in Cedar Key, the hypotheses for the forage network would be similar to the socialize network. However, inshore dolphins that feed predominantly on non-schooling fishes may experience relatively more feeding competition [36], thus the forage network may be significantly different from the socialize network. The forage network may indicate preferential associations among individuals (as seen in [23]) who have few weak associations with others and as a result forage in small, weakly connected groups. This also means that unlike the socialize network, dolphins that are connected to a particular dolphin are not expected to be connected to each other.
— *Overall network.* Because the overall network does not take behaviour into account but is built from all of the sightings, it should demonstrate some of the properties of each of the behavioural networks, but will not accurately represent any particular one.

## 3. Material and methods

### 3.1 Data collection

There are approximately 300 bottlenose dolphins (*T. truncatus*) that inhabit the general area of Cedar Key, Florida (29.0549° N, 83.0358° W), and many of them (approx. 250) are permanent residents [43,44]. Most of our study was carried out in shallow waters (1–10 m deep) where dolphin behaviours are readily observed. We collected data on the behavioural states of 147 resident bottlenose dolphins in Cedar Key over two different periods: from July 2008 through to December 2008 and from April 2010 through to August 2010. When a sighting of dolphins was encountered on a transect survey [45] or opportunistically [46], a slow approach was initiated. An assessment was made of the predominant behavioural state, defined as the activity of 50% or more of the individuals within the first 5 min of encounter [47–49]. Membership in each sighting was defined by the presence of dolphins during the first 5 min of encounter and within the 10 m chain rule [46]. Individual dolphins were photographically identified by comparing the markings on their dorsal fins and bodies [50,51] with those from an established catalogue [44,52]. If at any point during the approach or during the sighting, the dolphins changed their behaviour to avoid the research vessel or interact with it (e.g. if they attempted to bow ride), the sighting was excluded from our analysis.

Occasionally, sightings of dolphin groups of the same or similar composition as those previously sighted during a day were re-encountered. The dynamic nature of dolphin grouping decreases non-independent sampling problems; however we conservatively excluded sightings if any member had been sighted less than an hour previously, or if all of the members of the sighting were already sighted that day [46].

The behavioural states relevant to this study are: *socializing*, characterized by repeated incidents of body contact such as rubbing and petting with no consistent direction of movement [52–54]; *travelling*, characterized by spatial progress that is largely regular in terms of speed and consistent in terms of direction [52]; and *foraging*, characterized by prey capture or persistent incidents of prey searching as indicated by long dives or specialized feeding behaviours with direction shifts between surfacings [44,52].

The sightings used in the analysis cover 124 sampling days of the study and included 303 sighted groups. The average proportion of identified individuals per group was 0.80 (s.d. 0.21, min proportion 0.2, max proportion 1.0). The average number of sightings per individual was 4.29 (s.d. 4.39, min 1, max 21). As dependent calves that stay with their mothers and do not forage themselves could bias network associations, they were not included in the analyses.

### 3.2 Network construction

The dolphin sightings data resulted in four networks: an *overall network* that does not take behaviour into account, and the *socialize network*, the *travel network* and the *forage network* that correspond to their respective behaviours. Each sighting contributed vertices corresponding to dolphins in the sighting and an edge connecting each pair of vertices. The number of times that each dolphin was seen across all sightings was recorded as an attribute of the corresponding vertex. For a specified activity type, this construction resulted in a network that was: simple (i.e. no multi-edges or self-edges); undirected (i.e. if A is a neighbour of B then B is a neighbour of A) and weighted, with the weights being the half-weight index (*HWI*)=*the* number of times dolphins A and B were seen together divided by the total number of times they were seen together plus half the value of when A was seen without B and B was seen without A, and range from 0 for individuals that were never sighted together in groups to 1 for individuals that were always sighted together [55]. The HWI is commonly employed in dolphin studies [14], which facilitates comparison [27]. It should be noted that by construction, the edge weights in the networks are unaffected by variations in the average group sizes in the sightings data.

Following Whitehead [56], we pruned the networks by removing the vertices corresponding to dolphins sighted fewer than the threshold value (three in our study) at which the largest number of dolphins would be included in the networks while still allowing significant patterns of association (see the Dryad Digital Repository: http://dx.doi.org/10.5061/dryad.4r668).

Having a low threshold for inclusion, or simply including all available association data for all individuals, maximizes the information displayed but increases the sensitivity of the network to seldom-sighted and transient individuals. This may mask or confuse underlying social structure, thus limiting the ability of network analysis to decipher such structures, one of the main benefits of the technique. By contrast, having a high threshold for inclusion, while increasing confidence in displayed associations, decreases the detail of the network. This limits description of the network's overall structure and information regarding interconnections between distal elements of the network. Wey *et al.* [4] showed that network parameters are robust to different sampling efforts, and removal trials on simulated data have shown that the standard error within each trial was low, meaning the network parameters were measured precisely for different sample sizes [57]. A threshold of three sightings has also been used in other studies in dolphins [58] and in male zebras [39].

### 3.3 Network analysis

Network metrics that are pertinent to testing our hypotheses regarding the four networks (overall, socialize, travel and forage) are listed in table 1 along with definitions of the metrics, their biological significance in the context of the behavioural networks of dolphins and references to articles where more details on the metrics can be found.

**Table 1.** Definitions of network metrics, their biological significance in the context of the behavioural networks of dolphins and relevant references.

network concept	definition	biological significance	reference(s)
average degree	the degree of a vertex is the number of edges incident on it. The average degree of a network is the average value taken over all the vertices in the network	high average degree means each dolphin on average interacts with many other dolphins	[20]
average strength	the strength of a vertex is the sum of the weights of the edges incident on it. The average strength of a network is the average value over all the vertices in the network	high average strength means each dolphin on average interacts strongly with its neighbours	[59]
average edge weight	the average edge weight of a network is the average value of the edge weights over all the edges in the network	high average edge weight means that on average each pair of dolphins that interact with one another do so strongly. We used HWI values as edge weights	[20,59]
number of connected components	the total number of components, where each component is a set of vertices that are linked to each other by paths	large number of connected components means that there is a large number of dolphins with possible associations within the component they are in but no associations across	[20]
average clustering coefficient	the clustering coefficient of a vertex is the ratio of the number of edges between the vertices connected to it to the number of edges that could possibly exist between them. The average clustering coefficient of a network is the average value over all vertices in the network	large average clustering coefficient means pairs of dolphins that interact with a particular dolphin are likely to interact with one another	[8,20,59]
number of communities	the total number of communities where each community is a collection of vertices that are highly connected among themselves but with few or weak edges to vertices outside the collection. Communities within a network can be identified using the WalkTrap algorithm, which is based on the fact that a random walker tends to get trapped in dense parts of a network corresponding to communities	large number of communities means large number of groups of dolphins with strong intra-group connections and weak inter-group connections	[19,60]
average community size	the average number of vertices in a community	large average community size means each community on average has many dolphins with connections among themselves	[19]
community overlap	the distance between the partitions representing communities in networks, measured as the variation of information or shared information distance between the partitions	large community overlap means that dolphins that tend to associate closely with each other in one network also associate closely in the other	[61]

We tested for preferential associations among dolphins in each network using a modified permutation test against a null hypothesis that the dolphins associate randomly [62,63]. This test was performed using the compiled SocProg package 2.4 (available at http://myweb.dal.ca/~hwhitehe/social.htm; [64]) with 200 000 permutations per network.

We used a randomization test to evaluate the statistical significance of network measures. The null hypothesis is that a structural measure on the real network is no different from that of a random network. We accepted or rejected the null hypothesis by comparing the observed measure with the frequency distribution of the measure calculated for an ensemble of 10 000 random networks, each generated using edge rearrangement (see the Dryad Digital Repository: http://dx.doi.org/10.5061/dryad.4r668) [65,66].

We used the Mann–Whitney *U*-test to test for possible pairwise differences in group sizes [1]. Each network was compared with the others.

## 4. Results

Analysis using SocProg shows that there are preferential associations between individuals in the overall network, the socialize network and the travel network, but not in the forage network (table 2). This is not an artefact of sample size: the number of sightings in the forage network (153) is greater than that in the travel network (77) and socialize network (38).

**Table 2.** Results from SocProg [64] analysis of preferential associations among dolphins in the overall network, the socialize network, the travel network and the forage network, using an inclusion threshold of three sightings. (Realvalues are compared with random values (permuted 200 000 times per network). The mean, standard deviation (s.d.) and coefficient of variation (CV) of the HWI values are shown along with the *p*-value indicating whether the associations are significant. Values in italics indicate significant preferential associations.)

		association indices		
		real	random	*p*-value
overall network	mean	0.03312	0.03558	*0*.*00055*
	s.d.	0.08157	0.06967	0.99999
	CV	2.46307	1.95818	1
forage network	mean	0.04186	0.04181	0.53489
	s.d.	0.11154	0.09057	0.99999
	CV	2.66463	2.16634	0.99999
socialize network	mean	0.18217	0.18762	*0*.*03554*
	s.d.	0.20699	0.17423	1
	CV	1.13628	0.92884	0.99999
travel network	mean	0.0596	0.06184	*0*.*0033*
	s.d.	0.1317	0.11237	0.99999
	CV	2.20953	1.81724	1

The main characteristics of the four networks are listed in table 3. In the socialize network, individuals have strong and repeated connections to many other individuals (highest average degree, highest average strength, highest average edge weight). Socializing happens in large groups (largest group size per sighting, highest size per community), and these groups are not exclusive (least number of communities, fewest connected components). Dolphins that are connected to a particular dolphin are more likely to be connected to one another (highest clustering coefficient).

**Table 3.** Basic network quantities for the overall network, the socialize network, the travel network and the forage network. (Mann–Whitney *U*-tests of group size indicated significant differences in group size between each pair of networks (italics, *p*-value<0.003) except for travel to overall (*p*-value>0.562). Metrics (average degree, average strength, average edge weight, number of connected components, average clustering coefficient, number of communities, average community size) were tested using an edge rearrangement randomization test. Values in italics are statistically significant (*p*-value<0.003).)

	number of sightings	number of vertices	number of edges	average group size	average degree	average strength	average edge weight	number of connected components	average clustering coefficient	number of communities	average community size
overall network	303	147	2088	*3.991* (s.d. 3.913)	*28*	*4.835*	*0.170*	*1*	*0.568*	*18*	*8.167*
socialize network	38	42	458	*8.359* (s.d. 5.942)	*21*	*7.469*	*0.342*	*1*	*0.761*	4	10.5
travel network	77	53	302	*4.137* (s.d. 3.099)	*11*	*3.099*	*0.272*	*2*	*0.555*	*8*	6.625
forage network	153	76	462	*2.955* (s.d. 2.993)	*12*	3.140	*0.258*	*4*	*0.539*	*17*	*4.471*

Dolphins in the travel network do not have strong and repeated connections to many others except their preferential associates (lower average degree, lower average strength, lower average edge weight). Travelling happens in smaller groups than socializing (smaller group sizes per sighting, smaller community size, larger number of communities, larger number of connected components). Dolphins that are connected to a particular dolphin are less likely to be connected to each other (smaller clustering coefficient).

The travel network is comparable with the forage network in terms of its average strength and clustering coefficient. In many other aspects, such as average degree, number of connected components and number of communities, the travel network is intermediate between the socialize network and the forage network. There is no significant difference in group size between the overall network and the travel network (table 3). This indicates that though dolphins do have preferential associations while travelling, they do not travel in groups as large as those they socialize in or as small as they forage in.

Among the three activity networks, the dolphins in the forage network have the weakest and least repeated connections to other individuals (lower average degree, lower average strength, lowest average edge weight). Foraging happens in smaller groups than any other activity (smallest group sizes per sighting, smallest community size), and these groups are exclusive with fewer links to other foraging groups (highest number of communities) or they are more likely in groups that never forage together (highest number of connected components). Foraging dolphins that are connected to other foraging dolphins are not as likely to be connected to each other (lowest clustering coefficient) as they are in the socialize and forage networks.

A large community overlap between two networks means dolphins that tend to associate closely with each other in one network also associate closely in the other. Among the three activity networks, the socialize network and the travel network have the most substantial community structure overlap (table 4), the travel network and the forage network have the least and the socialize network and the forage network have an intermediate value. The overlap between the overall network and each activity network is less than that of the activity networks to each other.

**Table 4.** Pairwise community structure overlap for the overall network, the socialize network, the travel network and the forage network. (The smaller the numeric value, the larger the overlap.)

	overall network	socialize network	travel network	forage network
overall network	0	4.908	5.382	5.534
socialize network	4.908	0	3.031	4.686
travel network	5.382	3.031	0	5.278
forage network	5.534	4.686	5.278	0

## 5. Discussion

The results of our study provide clear evidence that the patterns of spatial associations among individuals differ depending on the behavioural state under consideration (table 2). We thus reject our null hypothesis that the four networks are similar to each other. As mentioned earlier, fission–fusion societies are often a response to the competing needs of social interactions (predator protection, social affiliations) and resource availability [25,67], and this should be seen in network analysis by behaviour. These differences are effectively captured through appropriate network analysis, as we have shown here (table 3). The overall network masks the differences that are seen in the networks sorted by behaviour (table 3). Using an overall network to describe a population also loses the important information gained by an analysis of community structure overlap. Namely, dolphins that tend to associate closely with each other in the socialize network also associate closely in the travel network, there is intermediate overlap of association between the forage and socialize networks and less so between the forage and travel networks (table 4). Important network properties that change according to the activity type considered include the average degree and average strength of vertices, the average edge weight (HWI), the number of communities, the average size of communities and the average clustering coefficient.

The values of the average clustering coefficients for the three activity networks show that there is a substantially greater likelihood that two dolphins which interact with a common third dolphin will also interact with each other in the socializing behavioural state than in either the travelling or foraging states. Dolphins engage in strong and frequent associations when socializing, but not when foraging (table 3). The strength and frequency of associations when dolphins are travelling is intermediate between that found when they are socializing or foraging.

These results show that a highly mobile species with extensive fission–fusion relationships may engage in certain inter-individual associations in some behavioural states but not in others. The cost–benefit ratio of interacting with an individual may vary with behavioural state. This may explain some of the features of the forage network. Bottlenose dolphins in Cedar Key have been observed in small, weakly connected groups (small group size and high numbers of connected components, no preferential associations, table 2; average strength in forage is not significantly different than a random model, table 3). A likely explanation for such behaviour is that prey are distributed singly or in patches small enough that competition generally disfavours the formation of groups. Current evidence in primate research supports this theory (e.g. [67]). For example, red colobus monkeys that forage in larger groups have reduced foraging efficiency than smaller groups [68].

Connor [40] refers to non-mutualistic clusters of individuals as *aggregations*, not groups, and notes that smaller aggregations are more likely to resemble mutualistic groups in scale. Non-mutualistic group formation can include aggregations where food is concentrated [69]. Other systems have also shown a possible correlation between group structure and food availability. Patchy distributions of prey have been shown to increase rates of fission–fusion in humpback dolphins [70]. Female baboons have cyclical, qualitative changes in the strength of their associations that depend on resource availability. When food is more abundant, these females do not have strong affiliations of any kind and instead have only connections that are more representative of gregariousness [71]. Heithaus & Dill [72] showed that prey availability for bottlenose dolphins is greater in shallower waters. If this is the case in Cedar Key, then the network structure of the dolphins forms for different reasons than at other sites; Gero *et al.* [23] demonstrated that preferred associations are strongest when foraging or socializing in Shark Bay, Australia, and females maintain acquaintance-level associations across behaviours, whereas males maintain affiliate-type relationships. The Cedar Key forage network shows no evidence of preferred associations (table 2). There was little evidence of cooperative foraging during this study (the cooperative driver–barrier behaviour described by Gazda *et al.* [44] was observed infrequently). Since dolphins have relatively low costs of locomotion compared with other mammals [41], they may be more able to maximize grouping benefits that are behaviour-specific [23,40]. We predict that networks will show less change with behavioural state in species with higher costs of locomotion.

Reduction of predation risk is thought to be one of the major factors favouring association across behavioural states in many mammal species [67,72–75]. Heithaus [74] suggests that sharks greater than 3 m in length are the primary predatory threat to immature dolphins. Predation risk is poorly understood in Cedar Key; Quintana-Rizzo & Wells [43] mentions seeing a lone bull shark once during the year-long study in Cedar Key, but communication with local fishermen indicates the occasional presence of large sharks. A sufficiently low predation risk in Cedar Key, in contrast to Shark Bay, where over 70% of non-calf dolphins have shark bite scars [74], may allow foraging in smaller, less connected groups. Dolphins in Cedar Key are occasionally observed foraging in a localized area without obvious signs of interaction or association, but the proximity of other individuals may still reduce predation risk. Reassessing the nature of foraging to delineate situations in which dolphins are foraging in proximity to, but not interacting with, other individuals would require reconsidering the definition of association in the foraging behavioural state. Association in our study was based on a 10 m chain rule. *Local enhancement* [76,77] offers an explanation for situations in which dolphins are foraging in proximity to, but not interacting with, other individuals. Dolphins may approach and forage near individuals that are catching fish, irrespective of social affiliation. Playbacks of foraging vocalizing dolphins could be used to establish the fish-catch detection distance.

Dependent calves were excluded from the study, and the sexes and ages of the individuals of the population remain largely unknown. Further study of this population in a network context would benefit from this information. In dolphins, males and females have differing association patterns, and this would affect network structure [23].

In conclusion, we have shown that network analysis successfully captures important differences in the social structure of bottlenose dolphins across different behavioural states. Individuals do not generally maintain the same level of association in different activity networks, and the community structure determined by the network structure changes depending on the activity under consideration. In general, it may be important to account for behavioural states when conducting network-based studies of social animals with fission–fusion characteristics.

## Data accessibility

Supporting data and software can be accessed from the Dryad Digital repository (http://dx.doi.org/10.5061/dryad.4r668).

## Data

The file all_orig.csv contains all the dolphin sightings, and the files socialise_orig.csv, travel_orig.csv and forage_orig.csv contain the sightings for the socializing, travelling and foraging behaviours, respectively. The file all.gml represents the overall network, and the files socialise.gml, travel.gml and forage.gml represent the socialize, travel and forage networks, respectively, each constructed using a threshold value of three sightings.

## Software

csv2gml.py constructs a network from the sightings data using the specified value for threshold. The resulting network is saved as a GML file.analyze.py performs basic network analysis on the specified networks and prints the results to the screen. The analysis includes the calculation of the community structure overlap between network pairs and the calculation of basic network metrics for each network.graph_permutations.py is a module for performing randomization tests on networks in order to test the statistical significance of network metrics.Preferential associations were tested with SocProg package 2.4.Mann–Whitney *U*-tests were performed using SPSS Statistics 19.

## Software dependencies

Python (https://www.python.org/)

NumPy (http://www.numpy.org/)

NetworkX (https://networkx.github.io/)

Igraph (http://igraph.org/redirect.html)

SPSS Statistics 19 (http://www-01.ibm.com/software/analytics/spss/products/statistics/)

SocProg package 2.4 (http://myweb.dal.ca/~hwhitehe/social.htm; [64])
